# A Manual of Human Microscopic Anatomy

**Published:** 1861-09

**Authors:** 


ART. X.—*A Manual of Human Microscopic Anatomy.* By A. KÖLLIKER, Prof. of Anat. and Phys. in the University of Würzburg. With 249 Illustrations. 8vo., pp. 633. London, 1860.

THIS valuable work, in its original German edition or in its English and American translations, is so well known, and has been so often noticed by the journals of Europe and America, that commendation now is almost superfluous. The author informs us that the book which he now presents to the English medical public is, in the main, a condensed version of the second German edition (of 1855) of his "Handbuch der Gewebehlehre," but that every material addition that has been made to human microscopic anatomy, up to the present date, is found incorporated in it. The work is, therefore, brought well up to the third German edition, that of 1859.

He calls attention to the fact that at the time of the issue of this translation, there was no work in English scientific literature devoted wholly and exclusively to the detailed treatment of the textural anatomy of man, as at present known. The translators, Dr. George Buchanan and "another friend," neither of whom is mentioned in the title-page, the latter being either too modest or too much neglected to be even alluded to by name in the preface, have done their duty well, and have thus given to the profession, thanks to the publisher, a handsome work, which is worthy of a more extended acquaintance with its merits than was possible in the restricted circulation of its previous edition by the Sydenham Society of London.

A new translation, with new additions and new views of histology, constitute virtually a new book, which, as a work of reference, deserves a place in every medical library. Good books on human microscopic anatomy are scarce, and that of Prof. Kölliker is so well known that we have not deemed it more than necessary to mention the fact of an improved edition having been issued.

